# Perceptions, Practices and mHealth Readiness for Blood Pressure Self‐Monitoring Among Ghanaian Hypertensive Patients

**DOI:** 10.1155/ijhy/5512077

**Published:** 2026-07-23

**Authors:** Evelyn K. Ansah, Valencia Koomson, Jacques Kpodonu, Saviour Edem Vidzro, John Tetteh, Swithin M. Swaray, Alice Tang, John Kpodonu, Mark Tettey, Frank Edwin

**Affiliations:** ^1^ Institute of Health Research, University of Health and Allied Sciences, Ho, Ghana, uhas.edu.gh; ^2^ Department of Electrical and Computer Engineering, Tufts University, Medford, Massachusetts, USA, tufts.edu; ^3^ Department of Surgery, Division of Cardiac Surgery, Beth Israel Deaconess Medical Centre, Boston, Massachusetts, USA; ^4^ Department of Community Health, University of Ghana Medical School, University of Ghana, Accra, Ghana, ug.edu.gh; ^5^ National Cardiothoracic Centre, Korle-Bu Teaching Hospital, Accra, Ghana, kbth.gov.gh; ^6^ Department of Social and Behavioral Science, School of Public Health, College of Health Sciences, Accra, Ghana; ^7^ Department of Public Health and Community Medicine, Tufts University School of Medicine, Boston, Massachusetts, USA, tufts.edu; ^8^ Department of Medicine and Therapeutics, University of Ghana Medical School, University of Ghana, Accra, Ghana, ug.edu.gh; ^9^ Department of Surgery, University of Ghana Medical School, University of Ghana, Accra, Ghana, ug.edu.gh; ^10^ School of Medicine, University of Health and Allied Sciences, Ho, Ghana, uhas.edu.gh; ^11^ Cardiothoracic Centre, Ho Teaching Hospital, Ho, Ghana, hth.gov.gh

## Abstract

**Background:**

Hypertension is a critical global health issue, with self‐monitoring of blood pressure emerging as an essential tool for its management. In Ghana, clinic visits are mostly scheduled at 2‐3 monthly intervals, making it difficult to assess patient blood pressure control in between visits. The use of mobile health applications for the self‐monitoring of blood pressure among hypertensive patients is not well‐documented. Empowering patients through self‐monitoring of blood pressure along with feedback from an interactive mobile health application could potentially improve blood pressure control. To ensure that such applications are effective, patient knowledge, perceptions and practices with regard to hypertension must be taken into consideration in the development of the interactive mobile health application. This study investigated patient knowledge of hypertension and the prevalence and practice of self‐monitoring, as well as perceptions of hypertension and self‐monitoring of blood pressure among Ghanaian patients diagnosed with hypertension at two teaching hospitals.

**Methods:**

A cross‐sectional pilot study using a mixed‐methods approach was conducted among patients aged 30 years and above who have been newly diagnosed without comorbidities with hypertension (blood pressure levels exceeding 140/90 mmHg). The pilot study explored perceptions and practices of the patients with regard to self‐monitoring for blood pressure control prior to the introduction of a mobile health technology intervention. Newly diagnosed patients without comorbidities were specifically recruited so that the focus of the study would be solely on hypertension management, ensuring that participants’ perceptions, knowledge and self‐monitoring practices were not influenced or confounded by the concurrent management of other medical conditions. Recruiting patients at the point of diagnosis also ensured that baseline assessments were conducted prior to any formal patient education or structured self‐monitoring coaching by healthcare providers, thereby providing an uncontaminated preintervention baseline for the planned mobile health intervention. Pregnant women and individuals with pre‐existing hypertension and coexisting medical conditions were excluded. Quantitative data were analysed using STATA Version 16, while qualitative data from in‐depth interviews were transcribed and analysed using NVivo Version 12. Themes from the qualitative analysis were used to contextualize the quantitative results.

**Results:**

The study included 32 hypertensive patients, with more males [53.1% (17/32)] than females. Overall, 71.9% (23/32) of patients knew normal and abnormal blood pressure values, with only 59.4% (19/32) demonstrating good general knowledge of hypertension. Notably, only 37.5% (12/32) of patients indicated that they routinely measured their blood pressure at home. Of the 12 respondents who did so, only nine ever recorded their blood pressure readings. Altogether, 62.5% (20/32) of respondents indicated that they felt comfortable measuring their blood pressure in the presence of others. With regard to confidence in using the blood pressure device independently, 59.4% (19/32) admitted to lacking confidence. Overall, 87.5% (28/32) indicated that they were comfortable using general smartphone applications with 56.2% (18/32) of respondents admitting to using phones for tracking their health status.

**Conclusions:**

This study highlights the need for enhanced information, education and communication if Ghanaian patients are to adopt self‐monitoring of their blood pressure in between clinic visits to take charge of their own health and improve hypertension control. Understanding these perceptions and knowing what the existing practices are is critical for healthcare providers to support patients effectively in managing their blood pressure and for the introduction of policies that support and promote the use of mobile health technologies in managing hypertension.

## 1. Introduction

Hypertension is a major global health challenge, contributing significantly to morbidity and mortality worldwide. According to the World Health Organization (WHO), approximately 1.13 billion people globally suffer from hypertension, with a significant proportion residing in low‐ and middle‐income countries (LMICs), including Ghana [1]. In Ghana, hypertension is a prevalent noncommunicable disease, and effective management is crucial for reducing associated health risks such as stroke, heart disease and renal failure [2].

Self‐monitoring of blood pressure (SMBP) has emerged as an important strategy in the management of hypertension. It empowers patients by allowing them to track their BP regularly, leading to better adherence to treatment regimens and lifestyle modifications [3]. The use of BP monitoring devices at home can enhance patients’ understanding of their condition and encourage proactive management of their health [4].

With advancements in technology, mobile health (mHealth) applications (apps) have become increasingly popular tools for managing various health conditions, including hypertension. BP monitoring apps offer several advantages, such as ease of use, real‐time tracking, data storage and the ability to share health information with healthcare providers [5, 6]. These apps can provide reminders for medication, alerts for abnormal readings and insights into trends over time, which can facilitate more informed clinical decisions and personalized care [7].

Despite growing enthusiasm for mHealth interventions in LMICs, several persistent challenges undermine their effectiveness, particularly for hypertension management. Digital literacy and access remain significant barriers. In rural and underserved communities, many individuals, especially older adults, lack the digital skills or smartphones necessary to engage effectively with health apps [8, 9]. Moreover, intermittent internet connectivity and unreliable electricity supply further constrain sustained use of these technologies in resource‐poor settings [10].

In Ghana, the adoption of mHealth technologies is rapidly expanding, driven by high mobile phone penetration (113.1%) and widespread smartphone ownership (99.7%). Since 2004, over 22 mHealth projects have been implemented to enhance healthcare delivery, supported by increasing internet accessibility and digital health initiatives [11]. A randomized controlled trial by Bolade‐Folasade Dele‐Ojo et al. in Ghana and Nigeria demonstrated the effectiveness of mHealth interventions in improving hypertension outcomes. In the study, 225 hypertensive adults received twice‐weekly SMS messages containing educational and motivational content over six months. The intervention group experienced a significant reduction in the systolic BP of 18.7 mmHg compared to 3.9 mmHg in the control group, and a higher rate of BP control (44.3% vs. 24.8%) [12].

Another pilot study in Ghana and Nigeria assessed the impact of an mHealth app, which sent lifestyle reminders to hypertensive patients. Over a six‐month period, participants showed a significant increase in physical activity and improved fruit and vegetable consumption, particularly by Months 3 and 6, respectively. However, the effects on physical activity declined over time, highlighting challenges in maintaining long‐term engagement [13].

While a number of studies assessing health outcomes are well documented, the perceptions and practices of hypertensive patients regarding self‐monitoring of their BP and the use of BP monitoring apps are not so well‐documented. Understanding these perceptions is crucial for developing effective interventions and educational programs that can enhance the adoption and appropriate use of such technologies [14]. This study explores the perceptions and practices of self‐monitoring BP using a mHealth monitoring app among Ghanaian patients living with hypertension. Specifically, the study (i) assessed patient knowledge of hypertension and the sociodemographic factors associated with that knowledge; (ii) examined the prevalence and current practices of SMBP before the introduction of the mHealth intervention; (iii) explored patient perceptions of BP monitoring devices and mHealth applications; and (iv) identified barriers and facilitators to the adoption of such technologies.

By examining how patients perceive and utilize these apps, the study seeks to identify barriers and facilitators to their use, providing insights that can inform healthcare policy and improve hypertension management in Ghana.

## 2. Methods

### 2.1. Study Design and Setting

This pilot study forms part of a broader study to assess the effectiveness of the use of a mHealth application along with SMBP for hypertension control in two cardiothoracic clinics, specifically the National Cardiothoracic Center, at Korle Bu Teaching Hospital in Accra and the Cardiothoracic Center at Ho Teaching Hospital, Ghana.

The pilot study, which preceded the introduction of SMBP along with the use of an interactive mHealth intervention, focused on documenting the perceptions and practice of hypertension patients with regard to self‐monitoring and control of their BP at baseline. A mixed‐methods approach was used, with quantitative and qualitative data being collected concurrently. The quantitative tool was an interviewer‐administered questionnaire, while the qualitative arm employed in‐depth interviews (IDIs) using an interview guide among all patients recruited for the pilot study. The two datasets were triangulated in the interpretation and discussion of the results of the study.

### 2.2. Study Participants, Sample Size and Sampling Procedure

We enrolled a total of 32 individuals newly diagnosed with hypertension without comorbidities, recruiting 16 participants from each study site. Newly diagnosed patients without comorbidities served two purposes. First, excluding patients with coexisting medical conditions ensured that the study findings reflected the experience of managing hypertension in isolation, without the confounding influence of other diseases or their treatments on BP control, self‐monitoring behaviour and perceptions of mHealth technology. Second, recruiting patients at the point of diagnosis ensured that their baseline knowledge, perceptions and self‐monitoring practices had not yet been shaped by prolonged clinical management or structured patient education, thereby providing a clean preintervention baseline for the broader mHealth intervention study. It is acknowledged that these criteria collectively limited the overall sample size, as newly diagnosed hypertensive patients without comorbidities presenting within the required 3‐to‐6‐month diagnostic window represented a relatively small proportion of the hypertensive clinic population at both facilities during the data collection period.

We used a consecutive sampling approach, whereby all patients presenting to the outpatient departments (OPDs) of the two health facilities during the data collection period were screened for eligibility. Every patient who met the inclusion criteria was approached and invited to participate in the study. Recruitment continued sequentially until the required sample size of 32 participants was achieved. This approach minimized selection bias by ensuring that no eligible patients were selectively excluded during the recruitment period.

The eligible participants included individuals aged 30 years and above, with systolic and diastolic BP measurements exceeding 140/90 mmHg who had been diagnosed with hypertension by a clinician within the past 3 to 6 months.

Additionally, only patients who owned and had regular access to a smartphone were eligible for inclusion, and patients without smartphone access were therefore excluded. We excluded pregnant women since some pregnant women have pregnancy‐induced hypertension, which may require additional management beyond what is provided for essential hypertension. We also excluded those with comorbid conditions because this often leads to complications that may make the BP difficult to control and may give a mixed picture of the result of SMBP by patients and make it difficult to assess the impact of the intervention.

### 2.3. Data Collection Method/Techniques and Tools

Patients who reported to the outpatient’s department of both hospitals were approached after triaging if their BP was above normal values. Each patient was screened by asking a few questions to ensure that they met the inclusion criteria. The eligible patient was then informed about the study before being requested to provide their written consent. Structured interviews, which lasted less than 30 min, were interviewer administered in a private area away from the outpatient’s department after obtaining patient consent. Sociodemographic data of participants, including age, sex, marital status, level of education, religion and ethnicity, were collected. Quantitative data were collected using a structured questionnaire comprised of three different sections to elicit patients’ knowledge of hypertension, practices related to hypertension and use of mobile phone applications in general as well as any mobile application for hypertension management. Following the completion of the structured interview, all enrolled participants received standardized instructions on SMBP delivered by healthcare workers. Instructions covered: correct placement of the cuff on the nondominant upper arm; recommended measurement timing (morning before medication intake and in the evening, after 5 min of seated rest); the number of readings per session (two consecutive readings at least 1 min apart); and how to interpret and record the values obtained. Instructions were provided verbally and reinforced with a pictorial guide translated into the local language of each study site to ensure comprehension across all literacy levels.

The structured questionnaire was adapted from previously published studies on hypertension knowledge, self‐management practices and mHealth utilization and was reviewed by public health and cardiology experts for content validity. It was pretested among 10 hypertensive patients at a similar facility not included in the study to assess clarity, relevance and comprehension, and minor revisions were made accordingly.

Electronic data collection was carried out with data entered directly into REDCap. Qualitative data were collected using an IDI guide on a scheduled day following the day of recruitment at the patient’s earliest convenience. Interviews were recorded with the patient’s consent and subsequently transcribed verbatim. The use of an interview guide ensured consistency in the issues explored across interviews.

### 2.4. Quality Control

Quality control was ensured during the different aspects of the study. To ensure reliability, experienced research coordinators were trained by the principal investigators on the study protocol and data collection before the commencement of the study. During training, the questionnaire was translated into the local language of the study areas and back into English. This was to ensure that the context was not lost. The data collection instruments were pretested in a health facility with similar characteristics to the study facilities. Following pretesting, the tools were revised. During the fieldwork, the research coordinators were supervised and closely monitored to make sure that all ethical and scientific requirements of the study were adhered to.

### 2.5. Data Analysis

Following data collection, the data were obtained from REDCap in the excel format and exported into STATA Version 16 (STATA Corporation, College Station, Texas) for data analysis.

Patients’ knowledge and practices were assessed utilizing a variety of questions. Four yes/no questions were asked to assess knowledge of hypertension. To assess patients’ knowledge of hypertension, we utilized four variables: understanding medical words used at the clinic, knowledge of systolic and diastolic BP, knowledge of normal and abnormal BP readings and knowledge of the normal value of systolic blood pressure. Each variable was coded as 1 (Yes) or 2 (No). The cumulative scores of all four variables were then combined to create an overall knowledge variable. A correct response received 1 point, while an incorrect response received 0 points. The overall knowledge score ranged from 0 to 4. Participants’ total knowledge was classified as Ideal/Good if they received a score of 4, Moderate if they received a score of 2–3 and Poor if they received a score between 0 and 1.

Lastly, to assess patients’ practices with regard to hypertension control, we assessed four variables: patient self‐measurement of BP at home, patient’s expressed confidence in measuring their own BP and also recording the readings at home, perceived availability of time for conducting BP measurements at home, adherence to routine BP medications and maintenance of a healthy routine at home. The variables were coded as 1 (Yes) or 2 (No), and the combined scores of all four variables formed an integrated practice variable.

For the four practice‐related questions, all ‘Yes’ responses received 1 point with ‘No’ responses receiving 0 points. If they received a score of 4, participants’ practice was classified as Ideal/Good, Moderate if they received a score between 2 and 3 and Poor if they received a score between 0 and 1. Simple frequencies and percentages were used to describe all categorical variables.

All recorded interviews were transcribed verbatim into English to preserve participants’ original meanings and expressions. The transcripts were imported into NVivo Version 12 for data management and analysis. An inductive thematic analysis approach was employed. Two members of the research team independently read and reread the transcripts to familiarize themselves with the data and conducted initial open coding. Codes were compared and harmonized through discussion, and a codebook was developed and iteratively refined as analysis progressed. Emerging codes were grouped into categories and subsequently organized into overarching themes and subthemes. Data collection and analysis occurred concurrently, allowing for the continuous assessment of thematic saturation. Thematic saturation was considered achieved when no new codes or themes emerged after successive interviews, and subsequent interviews yielded repetitive information. The final themes informed the reporting of qualitative findings and were used to contextualize and explain the quantitative results, particularly in highlighting barriers and facilitators influencing hypertension management.

### 2.6. Ethical Considerations

Ethical approval was sought from the Ethical Review Committees of the College of Health Sciences of the University Ghana and the Research Ethics Committee (REC) of University of Health and Allied Sciences. Approval was also sought from the Institutional Review Board (IRB) of the Korle Bu Teaching Hospital. Permission was sought from the hospital management teams of both health facilities. Participants had the option to withdraw from the study at any point throughout the interview. Patients were assured that refusing to participate in the study would not affect their care at the health facilities in any way. They were also assured of confidentiality. Interviews took place in locations where privacy was not violated. Data were anonymized in REDCap by assigning unique IDs instead of names to all respondents. After the interviews and survey, participants were thanked for their time.

## 3. Results

### 3.1. Demographic Characteristics of Patients

This pilot study enrolled 32 patients in total. Table 1 shows the sociodemographic characteristics of the participants. Overall, 46.9% (15/32) were female. A large proportion of patients [40.6%; (13/32)] were aged over 50 years, while 6.3% (2/32) were aged between 31 and 35 years. The majority of the patients [68.8%; (22/32)], were married. Education up to tertiary level had been achieved by 53.1% (17/32) of the patients, 28.1% (9/32) had completed secondary school, while 12.5% (4/32) indicated that they had no formal education. Overall, 96.9% (31/32) of study participants professed Christianity. With regard to ethnicity, most patients [40.6%; (13/32)] were Ewes, from the main ethnic group in one of the regions where the study was carried out with 28.1% (9/32) and 25.0% being of Akan and Ga/Dangme ethnic origin, respectively. See Table 1.

**TABLE 1:** Sociodemographic characteristics of study participants by study facility (*N* = 32).

Variable	Frequency (*n* = 32)	Percentage (%)
Sex		
Male	17	53.1
Female	15	46.9
Age (years)		
31–35	2	6.3
36–40	5	15.6
41–45	6	18.8
46–50	6	18.8
50+	13	40.6
Marital status		
Single	10	31.3
Married	22	68.8
Education		
No formal education	2	6.3
Primary level	4	12.5
Secondary level	9	28.1
Tertiary/higher level	17	53.1
Ethnicity		
Akan	9	28.1
Ga/Dangme	8	25.0
Ewe	13	40.6
Frafra^∗^	2	6.25
Religion		
Christian	31	96.9
Islam	1	3.1

^∗^Frafra refers to a subethnic group predominantly found in the Upper East Region of Ghana.

### 3.2. Patient Use of Mobile Phones and Phone Applications

All patients in the study reported using smartphones. Overall, 12.5% (4/32) needed assistance to use their smartphones, while the same proportion felt fully comfortable using mobile applications. Despite widespread smartphone use, the affordability of data remained a significant concern, with 59.4% (19/32) of participants expressing worry about the cost of data. In terms of data privacy, the majority (75.0%; 24/32) felt safe storing their personal information on mobile applications, while 25.0% (8/32) expressed concerns. When asked about using their phones to track health, responses varied. While 12.5% (4/32) of respondents always used their phones to track their health, 15.6% (5/32) did so sometimes and 43.7% (14/32) never did. See Table 2.

**TABLE 2:** Mobile phone usage.

Variable	No.	%
Type of phone		
Smartphone	32	100.0
Analogue	0	0.00
Need assistance to use smartphone		
Yes	4	12.5
No	28	87.5
Feel comfortable using apps on phone		
Yes	28	87.5
No	4	12.5
Worried about cost of data bundle		
Yes	19	59.4
No	13	40.6
Feel safe about personal data on applications		
Yes	24	75.0
No	8	25.0
Use phone to track health		
Always	4	12.5
Very often	3	9.4
Sometimes	5	15.6
Rarely	6	18.8
Never	14	43.7

Among participants who reported using mobile applications for health‐related purposes, the most commonly cited applications included Google Fit and Samsung Health, used primarily for step counting, calorie tracking and general wellness monitoring. A smaller number of participants reported using commercially available BP logging applications, specifically Blood Pressure Monitor Family Lite and SmartBP, to record home readings between clinic visits. No participant reported prior use of a hypertension‐specific mHealth intervention application before enrolment in this study, and none of the applications cited had been formally recommended or prescribed by healthcare providers at either facility.

Patient experiences, perceptions and behaviours were explored regarding the use of smartphones and mobile applications for health‐related purposes. Thematic analysis of data on the use of smartphones and mobile applications for health‐related purposes revealed four key themes: (1) Smartphone Ownership and Digital Literacy (2) Concerns About Data Cost, (3) Perceptions of Data Privacy and (4) Use of Phones to Track Health.

#### 3.2.1. Smartphone Ownership and Digital Literacy

All patients reported owning and using a smartphone, with many attesting to confidently navigating basic functions and applications. Most said they did not require assistance to operate their devices, although a small number admitted to needing support, especially when first learning to use a new app.

Patients generally felt comfortable with phone usage and demonstrated a willingness to engage with mHealth tools when adequately supported.

*‘Only when I was setting it up for the first time, someone helped me’. (IDI, Patient 441)*

#### 3.2.2. Concerns About Data Cost

Despite widespread smartphone ownership, data affordability emerged as a significant concern. Quantitatively, 59.4% (19/32) of participants reported being worried about the cost of data bundles (Table 2). The qualitative findings provide deeper insights into this concern, revealing that patients were cautious about using mobile data unless absolutely necessary, particularly those on limited or fixed incomes. For example, one participant stated

*‘Data is expensive, so I use it only when I have to’. (IDI, Patient 457)*

*‘When it gets finished, I get worried because I’ll not have information and now that I’m on retirement, that’s my source of joy’ (IDI, Patient 442)*

#### 3.2.3. Perceptions of Data Privacy

Most patients reported that they felt safe storing their personal health information on mobile applications, although a few raised concerns about who might access their data.

*‘I was worried about who else can see the readings. But they said it’s secure’. (IDI, Patient 440)*

*‘I feel safe using it but nowadays because of the hackers and all that when I’m using it; I feel they may hack my information but aside that I’m ok with everything’. (IDI, Patient 442)*

### 3.3. Use of Phones to Track Health

There was wide variation in how often patients used their phones to monitor or record health information. Some used them regularly to track BP or medication intake, while others rarely or never used health features.

*‘I don’t really use the phone for health. Just calls and messages’. (IDI, Patient 440)*

*‘When I see some signs then I google to see. When I take the medications and I realise that it might be this medication, I google to see whether it’s one of the side effects and then I know it is that, so I relax’. (IDI, Patient 442)*

### 3.4. Knowledge of Hypertension Among Patients

Three key themes related to patients’ knowledge of hypertension from the data are as follows: (1) Difficulty in Understanding Medical Terms, (2) Desire to Learn and Seek Clarity and (3) Role of Family Support and Personal Initiative.

#### 3.4.1. Difficulty Understanding Medical Terms

Most participants expressed challenges with comprehending medical terminology used by healthcare providers. This theme was often associated with limited formal medical knowledge and the professional jargon used in clinical settings. Keywords such as *‘terms’, ‘difficult’* and *‘don’t understand’* were recurrent in interviews. Some patients said

*‘I do have a challenge because I am not a medical person, so it is not every terminology that I understand’. (IDI, Patient 458)*

*‘Sometimes they use their terms and you may not understand… it’s not all the time that they’ll give you the full meaning’. (IDI, Patient 436)*

Only 31.3% (10/32) of patients indicated that they understood or were familiar with medical terms used at the hypertension clinic. Most of these patients attributed this to a personal interest in the medical field or assistance from family members who are healthcare professionals. Some patients mentioned occasional difficulties in understanding medical terms, but they indicated that they usually seek clarification from healthcare providers or resort to online resources like worldwide Google to gain a better understanding. Overall, while there were some challenges expressed, most participants indicated a willingness to learn more.

#### 3.4.2. Desire to Learn and Seek Clarity

Despite challenges, most patients demonstrated a proactive attitude toward understanding their health conditions. They reported asking healthcare workers for clarification or using tools such as the internet, especially Google, to interpret unfamiliar information to know more about their condition. The keywords *‘ask’, ‘Google’, ‘clarify’* and *‘learn’* frequently appeared in this context. Some said

*‘I sometimes don’t understand their terms, so I ask them what they mean by what they say’. (IDI, Patient 460)*

*‘When I don’t understand, I draw the doctor’s attention for an explanation… I ask them for clarification to understand and they do that for me’. (IDI, Patient 440)*

*‘When I take the medications and feel any side effect, I realise that it might be this medication, I google to see whether it’s one of the side effects’. (IDI, Patient 442)*

#### 3.4.3. Role of Family Support and Personal Initiative

Several participants, particularly older adults, reported relying on family members, especially children or spouses who are healthcare professionals, for explanation and support. This demonstrates the importance of social support systems in navigating hypertension management. Some said

*‘My children are nurses, so they explain to me if I don’t understand anything’. (IDI, Patient 435)*

*‘Sometimes I screenshot the medication and tell [my doctor friends]… these are the medications they gave me. Can you tell me more of the medication’. (IDI, Patient 436)*

The majority of patients [68.8% (22/32)] interviewed at baseline were able to correctly identify systolic as the upper number and diastolic as the lower number. Others, however, expressed confusion or uncertainty about which number represented systolic or diastolic pressure. Also, 68.8% (22/32) of patients interviewed indicated that a normal systolic BP should be 120 mmHg. Overall, 40.6% (13/32) of patients interviewed had good knowledge of hypertension, 37.5% (12/32) had moderate knowledge, while the remaining 40.6% (7/32) had poor knowledge. See Table 3.

**TABLE 3:** Knowledge of hypertension among patients.

Variable	No.	%
Understand medical words used at the clinic		
Yes	10	31.3
No	22	68.8
Knowledge of what systolic and diastolic blood pressure are		
Yes	22	68.8
No	10	31.3
Knowledge of normal and abnormal blood pressure readings		
Yes	23	71.9
No	9	28.1
Normal value of systolic blood pressure		
80 mmHg	6	18.8
100 mmHg	4	12.5
120 mmHg	22	68.8

Data from the IDIs confirmed what was found in the structured interviews. Many patients demonstrated a clear understanding of BP readings, correctly identifying systolic pressure as the upper number and diastolic pressure as the lower number. However, some expressed confusion or uncertainty about these terms or lacked confidence in interpreting them. While a few participants could accurately articulate the differences, others admitted they were unsure and relied instead on observing whether both numbers were generally ‘high’ or ‘low’ to guide their interpretation. Some said

*‘Systolic is the upper number and diastolic is the lower number’. (IDI, Patient 461)*

*‘I think the systolic is the top and the down one is diastolic and the third one is the pulse’. (IDI, Patient 458)*

*‘I know there are two different readings but I can’t really tell which one is systolic or diastolic. I’m only able to notice when the number goes very high and when it goes very low’. (IDI, Patient 443)*

Most patients were able to describe what they considered to be normal BP values, often referencing 120/80 mmHg as the ideal range. Others described what they believed to be unhealthy readings, often citing numbers that they had learnt from health workers or personal research. While some participants displayed knowledge of what normal or abnormal BPs are, others admitted that their knowledge was limited. They said

*‘According to what I know… if the systolic is 120 and the diastolic is 80, that is the right one’. (IDI, Patient 436)*

*‘Healthy blood pressure, I think is 120/80 but it goes with age too’. (IDI, Patient 455)*

*‘Unhealthy is when you read above 160–140/120–140 thereabout’. (IDI, Patient 440)*

*‘I don’t really know much about it’. (IDI, Patient 437)*

### 3.5. Relationship Between Knowledge of Hypertension and Sociodemographic Factors

With regard to the relationship between sociodemographic characteristics and general knowledge about hypertension, patient’s sex was found to be significantly associated with what they knew about hypertension (*χ* ^2^ = 4.979; *p* = 0.026). More males had good knowledge of hypertension as compared to females. Age was also associated with knowledge of hypertension, and this was statistically significant (*χ* ^2^ = 12.685; *p* = 0.013). Patients aged 36–45 years were more likely to have good knowledge of the condition as compared to older patients. There was no association between patient knowledge of hypertension and their marital status (*χ* ^2^ = 0.002; *p* = 0.961) as well as their educational level (*χ* ^2^ = 6.638; *p* = 0.084), respectively. See Table 4.

**TABLE 4:** Bivariate analysis of knowledge of hypertension and sociodemographic characteristics.

Variable	32 (%)	Moderate/poor knowledge (%)	Good (ideal) knowledge (%)	*χ* ^2^	*p* value
Sex					
Male	17 (53.1)	7 (36.8)	10 (76.9)	4.979	0.026
Female	15 (46.8)	12 (63.2)	3 (23.1)		
Age					
31–35	2 (6.3)	0 (0.0)	2 (15.4)	12.685	0.013
36–40	5 (15.6)	1 (5.3)	4 (30.8)		
41–45	6 (18.8)	2 (10.5)	4 (30.8)		
46–50	6 (18.8)	5 (26.3)	1 (7.7)		
50+	13 (40.6)	11 (57.9)	2 (15.4)		
Marital status					
Single	10 (31.3)	6 (31.6)	4 (30.8)	0.002	0.961
Married	22 (68.8)	13 (68.4)	9 (69.2)		
Education					
No school	2 (6.3)	2 (10.5)	0 (0.0)	6.638	0.084
Primary school	4 (12.5)	4 (21.1)	0 (0.0)		
Secondary school	9 (28.1)	6 (31.6)	3 (23.1)		
Tertiary or higher	17 (53.1)	7 (36.8)	10 (76.9)		
Ethnicity					
Akan	9 (28.1)	6 (31.6)	3 (23.1)	2.023	0.568
Ga/Dangme	8 (25.0)	4 (21.1)	4 (30.8)		
Ewe	13 (40.6)	7 (36.8)	6 (46.2)		
Other (please indicate ethnicity)	2 (6.3)	2 (10.5)	0 (0.0)		

### 3.6. Patient Perception of Hypertension and Its Management

Overall, 65.6% (21/32) of patients felt that they had adequate time available to measure their BP at home. Contrary to this, 12.5% (4/32) indicated that they never or rarely had time and as such may be unable to do so.

Of the 32 respondents interviewed, 62.5% (20/32) indicated that they felt comfortable taking their BP measurements around family or friends. Only 9.4% (3/32) felt judged for having high BP. The majority of patients [59.4%; (19/32)] lacked confidence in their ability to take their BP accurately at home.

While all respondents believed that hypertension medication can improve BP, the majority [53.1%; (17/32)] were of the view that herbal medicines were equally or sometimes more effective than the orthodox medicine they were being given at the hypertension clinic. See Table 5.

**TABLE 5:** Patient perception of hypertension and its management.

Variable	No.	%
Feel comfortable taking your blood pressure measurements around your family or friends		
Yes	20	62.5
No	12	37.5
Feel judged as someone with high blood pressure		
Yes	3	9.4
No	29	90.6
Believe hypertension medication can improve blood pressure a		
Yes	32	100.0
No	0	0.0
Believe herbal medicine is equally or more effective than orthodox medicine		
Yes	17	53.1
No	15	46.9
Confident in measuring blood pressure at home		
Yes	13	40.6
No	19	59.4
Spare time available for conducting blood pressure measurements at home.		
Always	11	34.4
Very often	10	31.3
Sometimes	7	21.9
Rarely	2	6.3
Never	2	6.3

### 3.7. Patient Practices With Regard to SMBP

With regard to the patient’s practices prior to their enrolment in the pilot study, only 37.5% (12/32) of patients indicated that they measured their BP at home. Out of the 12 patients who measured their BP at home, 9 of them indicated that they were documenting the measurements. In all, 43.8% (14/32)] of patients did not engage in good healthy life practices such as a healthy diet, regular physical activity and weight management that helps to control hypertension. See Table 6.

**TABLE 6:** Practices of hypertensive patients.

Variable	No.	%
Measure blood pressure at home		
Yes	12	37.5
No	20	62.5
Record blood pressure readings		
Yes	9	28.1
No	23	71.9
Take blood pressure medications routinely		
Yes	22	68.8
No	10	31.3
Tried a healthy routine that was good for health		
Yes	14	43.8
No	18	56.3

Patients indicated diverse practices in monitoring their BP at home, with notable variations in routine and frequency. Three main themes emerged from the analysis: (1) Consistency of SMBP, (2) Procedure Followed When Using Blood Pressure Monitoring Device at Home, and (3) Documentation of Blood Pressure Readings at Home.

These quotes illustrate various practices and approaches patients adopted when using a BP machine, highlighting routines, techniques and the importance of consistency and record‐keeping, as well as challenges faced in maintaining regular monitoring.

### 3.8. Consistency of SMBP

Some patients integrate SMBP into their daily routine, while others only monitored their BPs reactively.

#### 3.8.1. Integration of Self‐Monitoring Into Daily Routine

To ensure consistency, many patients described incorporating BP monitoring into their daily routines, often citing the morning as the most common time they take readings. Keywords such as *‘every morning’, ‘before I eat’* and *‘routinely’* were frequently used.

Several participants emphasized the importance of maintaining consistency, checking at the same time each day, sitting quietly and recording the results to track trends over time. They said

*‘I take my blood pressure first thing in the morning before I eat. It makes it easier for me to monitor changes’. (IDI, Patient 436)*

*‘I record it in the morning and evening… I take the measurement three times and then record the various readings’. (IDI, Patient 440)*

Some patients also received guidance from nurses or clinics on how to properly use the BP monitor, including correct cuff placement and positioning. This instruction helped reinforce confidence in their self‐monitoring practices.

*‘I feel almost 100% confident… I know at times, the machine can read error and I have to remove it, that is when I ask [my husband] to help’. (IDI, Patient 442)*

#### 3.8.2. Reactive Monitoring Behaviour

While many patients practiced regular monitoring, a few reported that they only measured their BP occasionally or when they felt unwell, reflecting a more reactive approach. These individuals were not likely to record readings consistently or incorporate monitoring into a structured routine. Keywords like *‘sometimes’, ‘when I feel something is wrong’* or *‘occasionally’* were commonly used. Some said

*‘I only check my blood pressure when I think something might be wrong, I don’t check it every day’. (IDI, Patient 458)*

Some patients also noted barriers to routine monitoring, such as time constraints or low motivation, despite being aware of the benefits.

*‘The only thing is that at times I don’t have time for myself… sometimes I feel very reluctant’. (IDI, Patient 440)*

### 3.9. Procedure Followed When Using BP Monitoring Device at Home

Patients described specific routines or techniques they used, such as positioning and timing, which they believed contributed to accurate readings. Some emphasized the importance of following instructions from healthcare providers.

*‘I make sure to follow the instructions carefully each time I use the BP machine, putting my hand in the right position’. (IDI, Patient 460)*

*‘With the machine that I have, on the cover, the colours are on the side. They are green, yellow, and red. so immediately I check, it’ll indicate whether I’m in red with an arrow, or if it’s yellow, it shows’ (IDI, Patient 442)*

*‘I take it when they are around but when I realise there will be distractions, I just close the door and send them to the hall, my husband will be there and in case of anything, I’ll call him’. (IDI, Patient 443)*

Some patients raised concerns about operational difficulties, particularly in cuff placement or interpreting error messages on the machine. Some said

*‘I have trouble using the BP machine correctly sometimes. It takes a bit of expertise to put on the cuff by yourself’. (IDI, Patient 454)*

### 3.10. Documentation of BP Readings

The majority of patients did not document the BP they measured; their main aim for checking their BP was to determine whether their hypertension was under control or not.

*‘At times I don’t record. It is when I’m told to maybe record for us to monitor, then I’ll record’. (IDI, Patient 455)*

*‘I started, but along the line, I had stopped’. (IDI, Patient 440)*

The few who documented BP readings did so by writing them down in a notebook or diary. Others recorded only if they received instructions from health providers to do so in between clinic visits. They explained that they documented the readings to show them to their doctors at the next clinic visit.

*‘I log the values of my blood pressure to present to my doctor during visit’. (IDI, Patient 458)*

*‘I have a diary that I also input my daily readings… I record it into the diary’. (IDI, Patient 457)*

*‘I have an exercise book in which I record it. The date and then everything, I record in an exercise book’. (IDI, Patient 455)*

### 3.11. Patient Perception of Use of the BP Monitoring Device to Measure Their BP at Home

Patients expressed a variety of perceptions regarding the use of the BP monitoring device SMBP at home. Three key themes emerged as follows: (1) Empowerment and Convenience, (2) Concerns About Accuracy and (3) Reassurance and Awareness of BP Fluctuations.

#### 3.11.1. Empowerment and Convenience

Many patients described feeling empowered, independent and more in control of their health when using the BP monitor at home. They valued the convenience and flexibility of measuring BP without visiting health facilities. This allowed them to adjust their lifestyle or medication adherence based on real‐time feedback from the device. Common keywords included *‘control’, ‘peace of mind’, ‘convenient’* and *‘monitor myself’.*

*‘I take my blood pressure first thing in the morning before I eat. It makes it easier for me to monitor changes’. (IDI, Patient 463)*

*‘It gives me prerequisite knowledge about my health, my system or my health status’. (IDI, Patient 436)*

*‘It is very convenient to check. It is not a problem for me to check with the machine’. (IDI, Patient 440)*

*‘It makes you bold and you know… at any given period of time… you can help somebody who is having that same problem’. (IDI, Patient 435)*

#### 3.11.2. Concerns About Accuracy

Although overall perceptions were positive, some patients expressed initial doubts about the accuracy of home BP monitors. Confidence improved when they compared home readings with clinic values. Phrases such as *‘confidence’, ‘error’,* ‘concerned’ recurred in these narratives. They said:

*‘At times, when the machine is giving you low or average reading… I compare with what I monitor at the hospital to be sure’. (IDI, Patient 442)*

*‘I was initially concerned about the home blood pressure monitor’s accuracy. However, after comparing it with the clinic’s readings, I now have greater faith in it’.(IDI, Patient 458)*

#### 3.11.3. Reassurance and Awareness of Fluctuations

Several participants reported that self‐monitoring at home helped to reduce anxiety, especially when compared to readings taken in clinical settings, where the anxiety from being in the clinical environment known as *‘white-coat effect’* may result in elevation of BP. This is a well‐known phenomenon. They appreciated the ability to monitor themselves in a calm, familiar environment and became more aware of daily BP fluctuations, linking changes to diet, medication or physical activity. Some said

*‘I feel more comfortable and confident doing it in the house than at the hospital’. (IDI, Patient 436)*

*‘Before I had the BP equipment at home, I used to be anxious when I went to the doctor’. (IDI, Patient 440)*

*‘I’ve noticed how much it fluctuates throughout the day… I’ve been able to see the effects of drinking water, and even when I feel tired’. (Synthesized across IDIs 442, 457)*

A summary of all qualitative themes and illustrative participant quotes is provided in Supporting Table S1.

## 4. Discussion

This study sought to explore the perceptions and practices of SMBP and the use of mobile phone health applications among Ghanaian hypertensive patients. By examining how patients perceive SMBP at home and how they utilize BP monitoring devices, mobile phones and existing applications, the study sought to identify barriers and facilitators to their use, providing insights that can inform policy on hypertension management in Ghana with the ultimate aim of improving it. The key findings are discussed below with particular emphasis on patient empowerment, patient choice and ease of use as cross‐cutting themes with direct policy relevance for Ghana’s commitment to expanding access to affordable health technology for the management of chronic diseases including hypertension.

### 4.1. Knowledge of Hypertension Among Patients

The findings indicated a mixed level of understanding among patients regarding various aspects of hypertension.

A substantial proportion of patients (68.8%) in this study reported difficulty understanding medical terms used at the clinic, which can hinder effective self‐management and communication with healthcare providers. Limited health literacy has been identified as a major barrier to chronic disease management, including hypertension, as it affects patients’ ability to comprehend and adhere to treatment recommendations. A study on mHealth‐based health literacy interventions demonstrated that improving health literacy can enhance medication adherence among hypertensive patients, ultimately leading to better disease control. Therefore, it is essential to prioritize health literacy and invest in strategies that simplify and improve communication in LMICs, where health literacy rates are often lower compared to high‐income countries [15].

Similarly, a meta‐analysis showed that low health literacy was consistently associated with poorer health outcomes. Their study found that patients with low health literacy had worse outcomes across a variety of conditions, partly due to their difficulties in understanding health‐related information and following treatment plans. A study in Uganda found that patients with limited knowledge of hypertension terminology were less likely to engage in health‐promoting behaviours, such as medication adherence and lifestyle changes [16]. Enhancing patient education and simplifying medical communication therefore are crucial steps to bridge this gap [17]. A study from Kenya highlighted that simplifying health communication and tailoring educational materials in a culturally sensitive manner significantly improved patients’ ability to understand their condition, leading to better engagement in self‐care practices and improved hypertension outcomes [18]. Therefore, it is essential to prioritize health literacy and invest in strategies that simplify and improve communication in LMICs, where health literacy rates are often lower compared to high‐income countries.

Most patients (68.8%) in this study demonstrated knowledge of what systolic and diastolic BPs are. The study further revealed that 71.9% of patients knew the values for normal and abnormal BP readings. Specifically, 68.8% correctly identified 120 mmHg as the normal systolic BP value. This level of awareness is critical as understanding these terms and knowing what the measurements mean is fundamental to effective hypertension management. It enables patients to recognize when their BP is within a healthy range or when it requires medical attention. Knowledge of these values is essential for patients to effectively monitor and manage their hypertension at home [19].

Patients who are well‐informed about their condition are more likely to adhere to their treatment plans and engage in self‐monitoring of their BP. This was also found in a systematic review, which similarly found that educational interventions that increased patient knowledge about their conditions improved adherence to treatment and better health outcomes [20]. In LMICs, where access to healthcare resources is often limited, these findings are particularly significant. For example, a study in India demonstrated that when patients received tailored education about hypertension, their adherence to antihypertensive medications improved, leading to better BP control [21]. Similarly, a study in South Africa found that patients with better knowledge of hypertension were more likely to engage in self‐monitoring of their BP, which was associated with improved health outcomes [22].

The review emphasized that when patients understand their health conditions and the rationale behind their treatment plans, they are more likely to engage in behaviours that support their health, such as taking medications as prescribed and monitoring their symptoms.

In this pilot study, 59.4% of patients demonstrated good knowledge of hypertension. This finding is important as it highlights both the progress made in patient education and the areas that still require attention. When compared to other recent studies, this result is slightly lower but within the range of global findings. Several studies have shown that patient knowledge is crucial for effective disease management, particularly for chronic conditions like hypertension. For instance, a study by Esen et al. found that approximately 68.0% of hypertensive patients had adequate knowledge about their condition, which is slightly higher than 59.4% observed in this study. This difference is attributed to the fact that participants in the Esen et al. study were more exposed to health education or counselling on hypertension [23].

### 4.2. Practices of Hypertensive Patients

The practices of hypertensive patients play a crucial role in the management and control of hypertension. This study assessed the prestudy practices of patients concerning BP monitoring and lifestyle modifications, highlighting significant gaps that need to be addressed to improve health outcomes.

A small proportion of patients (37.5%) measured their BP at home, despite evidence suggesting that regular home monitoring is associated with better hypertension control [24]. This low rate of self‐monitoring for BP control could be attributed to a lack of awareness, confidence or resources needed for regular monitoring. A study by Esen et al. in Turkey found a slightly higher knowledge of 55% of hypertensive patients engaged in regular home monitoring. This higher rate was associated with increased patient education and the availability of home monitoring equipment, which were both emphasized as key factors in promoting home BP monitoring [23]. Another study by Karami et al. in Iran reported even higher rates of home BP monitoring (60%–70%), which was largely attributed to the integration of structured patient education, routine follow‐up by healthcare providers and the use of mHealth‐supported adherence strategies that reinforced regular self‐monitoring [15]. The rate of home BP monitoring observed in this study (37.5%) was substantially lower than that reported in other LMIC settings such as Turkey (55%) and Iran (60%–70%), but higher than rates reported in Ethiopia (8.93%), Pakistan (< 25% regular HBPM) and Yemen (≈22% overall home SMBP), highlighting the wide variation across LMICs that likely reflects differences in healthcare infrastructure, access to validated devices and the availability of structured patient education and follow‐up support. [15, 25–28]. In Ghana, patients may face financial barriers to purchasing BP monitors, limited structured counselling on home monitoring techniques and reduced follow‐up reinforcement by healthcare providers. Additionally, the relatively low confidence in self‐measurement observed in this study (59.4% lacking confidence) may further explain the reduced uptake. Cultural beliefs, including trust in herbal medicine reported by nearly half of participants, may also influence engagement with biomedical self‐monitoring practices. These findings suggest that beyond technological availability, successful implementation of home monitoring in LMICs requires structured patient training, device accessibility and culturally sensitive education strategies [25].

These studies also highlighted the fact that socioeconomic factors, including limited access to resources such as BP cuffs and lack of awareness about the benefits of home monitoring, remain barriers to home BP monitoring in many LMICs. A study in Ghana pointed out that economic constraints and lack of training on how to measure BP at home were significant obstacles for many patients, despite the acknowledged benefits of home BP monitoring for effective hypertension management [29].

In this study, among patients who measured their BP at home, only 28.1% recorded their readings. In contrast, a study conducted in a structured primary care setting found that approximately 45% of patients who regularly monitored their BP at home documented their measurements. This relatively higher documentation rate was attributed to explicit instructions from healthcare providers to keep written records, routine review of BP logs during clinic visits and patient awareness that documented readings directly informed clinical decision‐making. The study emphasized that consistent documentation is linked to improved BP management and facilitates more accurate communication with healthcare providers [23].

A positive finding in the present study was that 68.8% of patients reported routinely taking their prescribed antihypertensive medications. Similarly, a population‐based study by Eghbali et al. in Iran reported a slightly higher medication adherence rate of approximately 70%, noting that adherence was generally lower among younger patients and those with multiple comorbidities [30]. To enhance medication adherence, strategies such as patient reminders, simplified dosing regimens and the management of side effects are essential for improving compliance [31].

The current study also found that only 43.8% of patients had tried a routine beneficial for their health, while 56.3% had not engaged in such practices. Lifestyle modifications, including a healthy diet, regular physical activity and weight management, are essential components of hypertension management [32]. The low engagement in healthy life routines suggests a need for comprehensive lifestyle intervention programs tailored to the patient’s cultural and socioeconomic context. These programs should focus on educating patients about the benefits of a healthy lifestyle and providing support to implement these changes [33].

### 4.3. Patient Perception of Hypertension and Its Management

In the current study, 37.5% of respondents reported discomfort taking their BP in the presence of family or friends. Similarly, a 2022 community‐based study conducted in a primary care setting reported that approximately 35% of patients avoided measuring their BP in front of others due to privacy concerns or fear of judgement, a behaviour shown to negatively affect adherence to home BP monitoring [34].

According to previous research, a considerable proportion of patients experience social support, which may have a favourable impact on their adherence to BP self‐monitoring activities and medication adherence. The patient’s overall acceptance and awareness of hypertension may also be reflected in the ease with which the problem is discussed and managed [35].

The majority of respondents (90.6%) reported not feeling judged for having high BP, suggesting that there are few significant social barriers for patients with hypertension, which is a positive finding. Similarly, Keene’s research indicated that while overall stigma is low, approximately 15%–20% of patients, particularly younger individuals, still reported experiencing some level of perceived judgement about their condition [34]. A supportive social environment has been linked to better health outcomes, making it crucial for the effective management of diseases [36].

All respondents thought that taking medication for hypertension can lower BP, which shows great faith in medication‐based treatment. This result is consistent with worldwide patterns in which people acknowledge the effectiveness of prescription drugs in treating hypertension [37].

There was contention, however, regarding the relative efficacy of herbal medicine and conventional (Western) medicine. About 46.9% of respondents in this study preferred herbal therapies. This demonstrates the continued importance and faith in herbal treatment in the Ghanaian context. When creating educational and intervention programs, it is imperative to take these preferences into account because including culturally appropriate techniques can improve acceptability and adherence [38].

One interesting finding was that 59.4% of participants expressed low trust in their ability to take their BP at home. There are a few possible reasons for this lack of confidence, such as a lack of access to appropriate training, a fear of taking the wrong measurements or technical issues with the monitoring equipment [39]. Better patient education and the provision of user‐friendly devices may result in increased confidence in SMBP.

Patients’ perceptions of the availability of free time for taking their BP readings at home differed. While many patients were able to integrate BP monitoring into their daily routines, a considerable number of them had time constraints, according to these findings. Providing flexible monitoring choices and customizing BP self‐monitoring recommendations to meet individual schedules could improve adherence [24].

### 4.4. Patients’ Perceptions of Using BP Monitors at Home

The results of this study shed light on the diverse perceptions and experiences of patients regarding the use of BP machines at home. The patients’ views on using BP machines to measure their own BP at home revealed several key insights.

Many patients expressed a sense of empowerment and control over their health through the use of home BP monitors. This finding aligns with previous research indicating that SMBP can lead to improved patient engagement and adherence to treatment plans [38].

Patients appreciated the insight gained from monitoring their BP at home, particularly in understanding how lifestyle choices and medication adherence directly influence their readings. This aligns with studies suggesting that BP self‐monitoring can enhance patients’ awareness of their health status and motivate positive behaviour changes [4]. The daily fluctuations in BP observed at home also increased patients’ awareness of their condition, potentially leading to better management strategies.

Despite the benefits perceived by patients, concerns regarding the accuracy of home BP monitors were evident. Some patients initially doubted the reliability of the devices but gained confidence after validating their readings against clinic measurements. This highlights the importance of providing clear instructions and guidance to ensure accurate readings and alleviate patient apprehensions [40]. Another study in Ghana found that patients’ concerns about device accuracy were common, but those who received training on how to use the monitors and confirmation of the readings during clinic visits showed higher confidence in using home BP monitoring effectively [29]. Additionally, challenges related to using the device correctly, such as cuff placement, were reported by some patients, indicating the need for ongoing support and education.

The ability to take BP readings in a relaxed home environment was noted to reduce anxiety for many patients, contrasting with the stress often associated with clinical settings. This finding is consistent with research suggesting that home monitoring can lower white‐coat hypertension and improve patient comfort during BP measurements [41]. The convenience of home monitoring was particularly valued by patients with busy schedules, eliminating the need for frequent clinic visits and enabling timely monitoring of their BP.

### 4.5. Policy and Implementation Implications

Ghana’s high mobile phone penetration (113.1%) and widespread smartphone ownership present a significant opportunity to scale mHealth interventions for hypertension management nationally. The near‐universal smartphone use observed in this study suggests that technological access is no longer the primary barrier to digital health adoption. However, the finding that 59.4% of participants were concerned about data affordability highlights the need for cost‐sensitive implementation strategies. Policymakers could leverage Ghana’s digital infrastructure by integrating hypertension mHealth platforms into existing national eHealth frameworks while partnering with telecommunications providers to subsidize or zero‐rate health‐related data usage, similar to models used for educational and COVID‐19 information platforms. Additionally, incorporating USSD‐based or low‐bandwidth solutions may reduce reliance on continuous internet access, making interventions more inclusive for lower‐income users. Embedding mHealth tools within routine hypertension clinic services and linking them to community health workers could further enhance uptake and sustainability. Addressing affordability barriers while capitalizing on high smartphone penetration could significantly accelerate mHealth scaling and contribute to improved hypertension control in Ghana.

Recent LMIC‐focused studies published in cardiovascular and digital health journals have shown that mHealth interventions can significantly improve hypertension control when integrated into routine care with structured education and follow‐up support. For example, a randomized trial in Nigeria and Ghana found that a mobile phone–based messaging intervention significantly improved systolic BP reduction and control rates among patients with hypertension compared with usual care (SBP reduction −18.7 vs −3.9 mmHg; 44.3% vs 24.8% control) over 6 months, demonstrating the clinical impact of digital support in sub‐Saharan African settings [12]. Similarly, a multifaceted smartphone‐based mHealth intervention in a low‐to‐middle‐income country significantly enhanced medication adherence and reduced systolic BP over 6 months compared with standard care [42]. Such strategies align with Ghana’s commitment to achieving Universal Health Coverage under SDG 3 by improving access to affordable digital health services for chronic disease management.

Central to translating these findings into sustainable policy is the principle of patient empowerment. Participants in this study who engaged in home BP monitoring reported greater perceived control over their health, an improved ability to link their lifestyle behaviours to readings and reduced anxiety compared with clinic‐based measurement, all hallmarks of effective chronic disease self‐management. To realize these benefits at scale, mHealth tools for hypertension in Ghana must be designed with ease of use as a non‐negotiable criterion: intuitive interfaces, local language support, offline functionality and low data consumption are essential rather than optional features. Patient choice is equally critical. A nationally scaled mHealth policy should not mandate a single application but instead offer patients a validated range of options, from USSD‐based systems operable on basic handsets to feature‐rich smartphone applications, ensuring equity across literacy and income levels. Empowerment, choice and ease of use are therefore the three pillars on which Ghana can build a patient‐centred mHealth framework for hypertension that is clinically effective, equitable and aligned with its Universal Health Coverage.

### 4.6. Limitations

This pilot study has some limitations. The small sample size and recruitment from only two tertiary health facilities limit generalizability and may introduce selection bias, particularly for patients managed in primary or rural settings. It may not therefore fully represent the diverse experiences of hypertensive patients in Ghana. The cross‐sectional design prevents causal inference regarding the relationships among knowledge, self‐monitoring practices and health outcomes. Additionally, data on medication adherence, lifestyle practices and home BP monitoring were self‐reported and may be subject to social desirability bias, with participants potentially overstating adherence or positive health behaviours. This may have led to an overestimation of actual practice levels.

The exclusion of patients with coexisting medical conditions further limits applicability to more clinically complex hypertensive populations, whose self‐management experiences may differ. Nonetheless, the study offers valuable insights to inform the development and implementation of mHealth interventions for hypertension management in Ghana.

While the sample size was sufficient for pilot feasibility testing, larger studies are needed to validate findings across diverse populations. Future research should incorporate larger and longitudinal designs to better assess the long‐term impact of patient perceptions and self‐monitoring.

## 5. Conclusion

In conclusion, the varied perspectives shared by patients highlight the multifaceted nature of perceptions and practices regarding SMBP at home. Understanding these perceptions and knowing what the existing practices are is critical for healthcare providers to support patients effectively in managing their BP and for the introduction of policies that support and promote the use of mHealth technologies in managing hypertension.

## Author Contributions

Concept and design: Valencia Koomson, Mark Tettey, Evelyn K. Ansah, Frank Edwin, John Kpodonu and Jacques Kpodonu.

Supervision: Evelyn K. Ansah, Frank Edwin, Mark Tettey, Valencia Koomson, John Kpodonu and Jacques Kpodonu.

Data curation: Saviour Edem Vidzro, Evelyn K. Ansah, Frank Edwin, Swithin M. Swaray and John Tetteh.

Analysis and interpretation of data: Saviour Edem Vidzro, Evelyn K. Ansah, Frank Edwin, Swithin M. Swaray, John Tetteh and Alice Tang.

Methodology: Evelyn K. Ansah, Frank Edwin, Mark Tettey, Valencia Koomson, Alice Tang, Saviour Edem Vidzro, John Tetteh and Swithin M. Swaray.

Validation: Evelyn K. Ansah, Frank Edwin, Swithin M. Swaray, Saviour Edem Vidzro, John Tetteh, Alice Tang, Mark Tettey and Valencia Koomson.

Drafting of manuscript: Saviour Edem Vidzro, Evelyn K. Ansah, Swithin M. Swaray, John Tetteh, Frank Edwin and Mark Tettey.

Review and editing: Evelyn K. Ansah, Frank Edwin, Alice Tang, Valencia Koomson, Mark Tettey, John Kpodonu, Jacques Kpodonu, Saviour Edem Vidzro and Swithin M. Swaray.

## Funding

This work was supported by the National Institutes of Health (NIH) (Grant No. 1R21EB033166‐01).

## Ethics Statement

This study protocol was approved by the Ethical Review Committees of the College of Health Sciences, University Ghana ID: CHS‐E6/M.5‐P4.7/2021–2022, and the Research Ethics Committee (REC) of the University of Health and Allied Sciences, ID: UHAS‐REC A.6[1]21–22, as well as the Institutional Review Board (IRB) of the Korle Bu Teaching Hospital, ID: STC000212/2021. Additionally, permission to involve individuals was obtained from both health facilities. Study participants provided individual written informed consent prior to participation in this study. All participants were given comprehensive information about the study’s purpose during the recruitment process.

## Conflicts of Interest

The authors declare no conflicts of interest.

## Supporting Information

Additional supporting information can be found online in the Supporting Information section.

## Supporting information


**Supporting Information** Supporting Table S1. Summary of qualitative themes and illustrative quotes.

## Data Availability

The data that support the findings of this study are available on request from the corresponding author. The data are not publicly available due to privacy or ethical restrictions.
