# Supplementary material for: Perceptions, Practices and mHealth Readiness for Blood Pressure Self‐Monitoring Among Ghanaian Hypertensive Patients
Source: Int J Hypertens. 2026 Jul 23;2026:5512077. doi: 10.1155/ijhy/5512077 (PMC13396871; doi:10.1155/ijhy/5512077)
Supplement: Supplementary file 1 — Supporting Information Supporting Table S1. Summary of qualitative themes and illustrative quotes. [file IJHY-2026-5512077-s001.docx]

**Supplementary Table S1. Summary of Qualitative Themes and Illustrative Quotes**

| **Main Theme** | **Subtheme** | **Illustrative Quote** | **Participant ID** |
| --- | --- | --- | --- |
| **Smartphone Ownership & Digital Literacy** | Initial assistance | “Only when I was setting it up for the first time, someone helped me.” | IDI 441 |
|  | General comfort | “I feel safe using it but nowadays because of the hackers and all that when I’m using it; I feel they may hack my information but aside that I’m ok with everything.” | IDI 442 |
| **Concerns About Data Cost** | Data affordability | “Data is expensive, so I use it only when I have to.” | IDI 457 |
|  | Retirement/limited income | “When it gets finished, I get worried because I’ll not have information and now that I’m on retirement, that’s my source of joy.” | IDI 442 |
| **Perceptions of Data Privacy** | Security concerns | “I was worried about who else can see the readings. But they said it’s secure.” | IDI 440 |
|  | Fear of hacking | “I feel they may hack my information but aside that I’m ok with everything.” | IDI 442 |
| **Use of Phones to Track Health** | Limited use | “I don’t really use the phone for health. Just calls and messages.” | IDI 440 |
|  | Searching for side effects | “When I see some signs then I google… I know it is that, so I relax.” | IDI 442 |
| **Difficulty Understanding Medical Terms** | Limited comprehension | “I do have a challenge because I am not a medical person, so it is not every terminology that I understand.” | IDI 458 |
|  | Medical jargon | “Sometimes they use their terms and you may not understand…” | IDI 436 |
| **Desire to Learn and Seek Clarity** | Asking for clarification | “I sometimes don’t understand their terms, so I ask them what they mean…” | IDI 460 |
|  | Seeking explanation | “When I don’t understand, I draw the doctor’s attention…” | IDI 440 |
|  | Googling medications | “When I take the medications… I google to see whether it’s one of the side effects.” | IDI 442 |
| **Family Support & Personal Initiative** | Support from children | “My children are nurses, so they explain to me if I don’t understand anything.” | IDI 435 |
|  | Consulting doctor friends | “Sometimes I screenshot the medication and tell them…” | IDI 436 |
| **Understanding** blood pressure (**BP) Values** | Correct identification | “Systolic is the upper number and diastolic is the lower number.” | IDI 461 |
|  | Partial understanding | “I think the systolic is the top…” | IDI 458 |
|  | Uncertainty | “I can’t really tell which one is systolic or diastolic.” | IDI 443 |
|  | Ideal blood pressure (BP) value | “If the systolic is 120 and the diastolic is 80, that is the right one.” | IDI 436 |
|  | Age consideration | “Healthy blood pressure… it goes with age too.” | IDI 455 |
|  | Unhealthy range | “Unhealthy is when you read above 160–140…” | IDI 440 |
|  | Limited knowledge | “I don’t really know much about it.” | IDI 437 |
| **Consistency of Self-Monitoring** | Morning routine | “I take my blood pressure first thing in the morning…” | IDI 436 |
|  | Recording multiple readings | “I take the measurement three times and then record…” | IDI 440 |
|  | Diary documentation | “I have a diary that I input my daily readings.” | IDI 457 |
|  | Presenting to doctor | “I log the values… to present to my doctor.” | IDI 440 |
|  | Confidence in device | “I feel almost 100% confident…” | IDI 442 |
| **Reactive Monitoring Behaviour** | Occasional checking | “I only check my blood pressure when I think something might be wrong.” | IDI 458 |
|  | Time constraints | “Sometimes I don't have time… I feel very reluctant.” | IDI 440 |
| **Procedure for Using** blood pressure (**BP) Device** | Following instructions | “I make sure to follow the instructions carefully…” | IDI 460 |
|  | Color-coded indicator | “They are green, yellow, and red… it shows.” | IDI 442 |
|  | Avoiding distractions | “When I realise there will be distractions, I just close the door…” | IDI 443 |
|  | Difficulty with cuff | “It takes a bit of expertise to put on the cuff by yourself.” | IDI 454 |
| **Documentation Practices** | Conditional recording | “It is when I'm told to maybe record… then I'll record.” | IDI 455 |
|  | Stopped recording | “I started, but along the line, I had stopped.” | IDI 440 |
|  | Exercise book | “I have an exercise book in which I record it.” | IDI 455 |
| **Empowerment & Convenience** | Health awareness | “It gives me prerequisite knowledge about my health status.” | IDI 436 |
|  | Convenience | “It is very convenient to check.” | IDI 440 |
|  | Helping others | “You can help somebody who is having that same problem.” | IDI 435 |
| **Concerns About Accuracy** | Comparing readings | “I compare with what I monitor at the hospital…” | IDI 442 |
|  | Gaining trust | “After comparing it with the clinic's readings, I now have greater faith in it.” | IDI 458 |
| **Reassurance & White Coat Effect** | Comfort at home | “I feel more comfortable and confident doing it in the house…” | IDI 436 |
|  | Reduced anxiety | “Before I had the BP equipment at home, I used to be anxious…” | IDI 440 |
